# A Case of Kawasaki Disease Accompanied by Encephalitis and Several Kinds of Arrhythmia during the Acute Phase

**DOI:** 10.1155/2019/7358753

**Published:** 2019-10-20

**Authors:** Naomi Nakagawa, Masahiro Kamada, Yukiko Ishiguchi, Yuji Moritoh, Kengo Okamoto, Shinji Itamura

**Affiliations:** ^1^Hiroshima Citizens Hospital, Department of Pediatric Cardiology, Hiroshima, Japan; ^2^Hiroshima Citizens Hospital, Department of Pediatric Neurology, Hiroshima, Japan

## Abstract

Although central nervous system complications occasionally accompany during the acute phase of Kawasaki disease, clinically problematic arrhythmia is quite rare. We report a case accompanied by encephalitis and several kinds of problematic arrhythmia. Following the diagnosis of Taussig–Bing anomaly and coarctation of the aorta, the patient underwent aortic arch reconstruction, an arterial switch operation, and ventricular septal defect closure. No significant arrhythmias were observed. At the age of 5 years, the patient presented with a fever, rash, conjunctival hyperemia, and redness of the lips and fingertips. He was subsequently diagnosed with Kawasaki disease. The patient also presented with disorientation, and electroencephalography revealed overall slow-wave activity, indicating encephalitis. The patient received high-dose immunoglobulin and steroid pulse therapy. Sinus arrest was detected on day 10, and an atrial flutter with a 2 : 1 to 4 : 1 atrioventricular conduction block occurred on day 20. Although cardioversion succeeded in alleviating the atrial flatter, the patient experienced significant sinus arrest. The sinus arrest was alleviated 3 days later. Kawasaki disease-induced vasculitis and the arterial switch operation may both have influenced the sinus node dysfunction. Although sinus node function recovered, the possibility of progression into the sinus node dysfunction in the future should be considered.

## 1. Introduction

Kawasaki disease (KD) is acute vasculitis of unknown etiology that was first described by Dr. Tomisaku Kawasaki in 1967 [1]. The most common sites of end-organ damage are the coronary arteries; however, an inflammatory response is induced in medium and small vessels throughout the body. Furthermore, complications of the central nervous system (CNS), including convulsions, disturbance of consciousness, aseptic meningitis, encephalopathy, and facial palsy, have been reported in patients with KD although CNS complications are uncommon in KD [2–4].

Arrhythmia severe enough to be clinically problematic during the acute phase of KD is rare [5] although pancarditis including the inflammation of the conduction systems in KD has been reported [6]. This case report describes the postoperative case of a newborn with Taussig–Bing anomaly with coarctation of the aorta who experienced encephalitis and various types of problematic arrhythmias during the acute phase of KD.

## 2. Case Report

One day after birth, the male newborn was transferred to Hiroshima Citizen's Hospital in a state of shock and was diagnosed with Taussig–Bing anomaly with coarctation of the aorta. After maintenance therapy for shock recovery, the patient, aged 4 days, underwent aortic arch reconstruction and pulmonary arterial banding. After coronary angiography which revealed that his coronary arteries were Shaher type 1, he underwent an arterial switch operation and ventricular septal defect closure at the age of 2 months. As his pulmonary branch stenosis progressed severely, the patient underwent percutaneous transluminal pulmonary angioplasty followed by surgical plasty of the left pulmonary artery at the age of 2 years. No significant arrhythmias were detected before or after these surgeries.

At the age of 5 years 6 months, the patient developed antibiotic-resistant fever. Six days later, he was admitted to Hiroshima Citizen's Hospital with exanthema, conjunctival hyperemia, redness of the lips, strawberry tongue, and erythema of the fingertips. On admission, he had a temperature of 37.9°C, a pulse of 141/min, and a blood pressure of 117/72 mm Hg. Both lung fields were clear on auscultation. The patient's heart rhythm was regular with a systolic murmur of Levine IV/VI, which was consistent with previous findings. Both his cervical lymph nodes were swollen and painful. The patient was expressionless with a consciousness level of E4V3M5, as determined by the Glasgow Coma Scale.

Laboratory blood tests performed at admission demonstrated the following: white blood cell count, 3,300/*μ*L; hemoglobin, 13.5 g/dl; hematocrit, 0.38; platelets, 81 × 10^9^/mm^3^; C-reactive protein, 6.6 mg/dl; sodium, 133 mEq/L; aspartate aminotransferase, 186 IU/L; alanine aminotransferase, 137 IU/L; and serum albumin, 3.7 g/dl. Examination of the patient's cerebrospinal fluid (CSF) demonstrated a glucose level of 57 mg/dl, protein level of 28 mg/dl, and a white blood cell count of 3/*μ*L.

**Figure 1:**
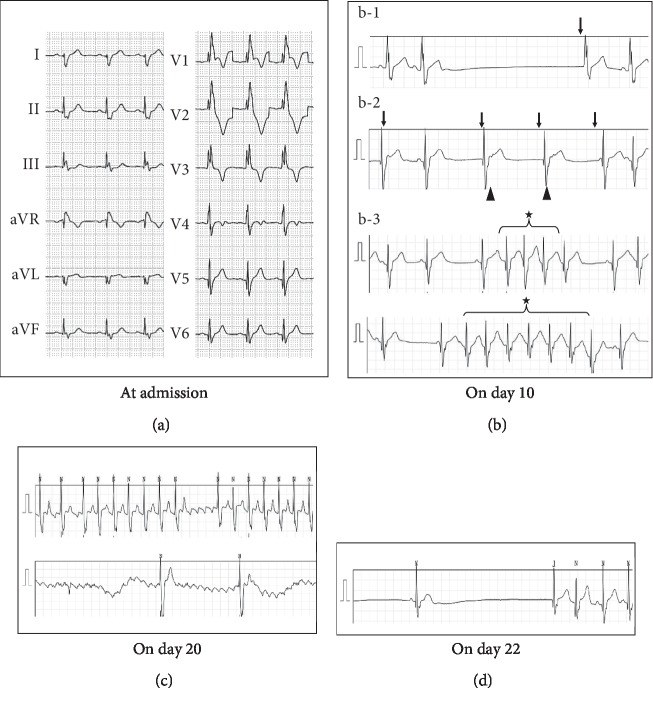
Electrocardiograms (a). Sinus arrest was detected (b-1), junctional rhythms (↓) were pointed with retrograde P waves (▲), and short bursts of atrial tachycardia were detected (★). Atrial flutter occured with a resultant 2 : 1 to 4 : 1 atrioventricular conduction block (c). Sinus arrest occured (maximum, 5.7 s) (d).

A standard 12-lead electrocardiogram (ECG) was performed at admission, which demonstrated a regular sinus rhythm and complete right bundle branch block, which was consistent with the findings of a previous ECG (Figure 1(a)). A chest roentgenogram demonstrated mild cardiomegaly (cardio thoracic ratio, 0.60), which was also consistent with previous results, without pleural effusion. An echocardiogram demonstrated the following: left ventricle end diastolic dimension, 33.6 mm (105% of normal range); left ventricle end systolic dimension, 18.8 mm; left ventricle ejection fraction (LVEF) and fractioning shortening (SF), 83% and 44%, respectively; mild mitral regurgitation; mild tricuspid regurgitation; mild pulmonary regurgitation; and pulmonary stenosis, 3.6 ms/s, without coronary arterial lesions and pericardial effusion. No significant differences were detected between these results and previous echocardiogram findings.

**Figure 2:**
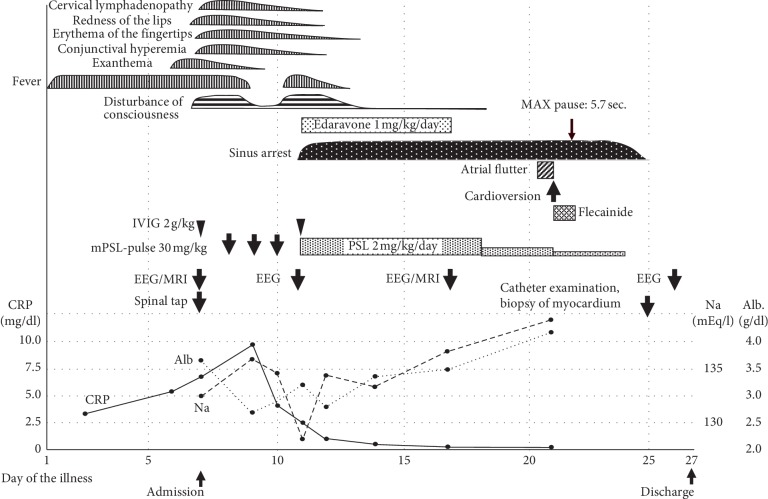
Clinical course.

Electroencephalogram (EEG) indicated diffuse disorganized high-voltage slow waves. Magnetic resonance imaging (MRI) at admission detected mild cerebral edema. The patient was diagnosed with KD-associated encephalitis and subsequently received methylprednisolone pulse therapy (30 mg/kg for 3 days), high-dose intravenous immunoglobulin (IVIG, 2 g/kg), and flurbiprofen (5 mg/kg/day). Consequently, his fever resolved, his consciousness level improved, and the other KD symptoms were also resolved. However, the fever recurred on day 10 of the illness, and the patient's level of consciousness deteriorated; therefore, additional 2 g/kg of IVIG and edaravone (1 mg/kg/day) were administered (Figure 2).

Abnormal ECG findings were subsequently detected (Figure 1(b)). Sinus arrest and junctional escape rhythm were detected on day 10 of the illness, followed by a short burst of atrial tachycardia. The patient was not administered any specific treatment for arrhythmia, as the sinus arrest had no significant influence on hemodynamics.

Mild pleural effusion was detected on the left side on day 12 of the illness; however, no novel abnormal findings were detected on echocardiography, including pericardial effusion. He regained his consciousness completely, and EEG showed normal findings on day 17. An oral steroid, edaravone, was administered for 7 days, after which the dosage was gradually reduced and discontinued.

Although the LVEF declined to 72% on day 9 transiently, it improved over 80% on day 15 of the illness and there was no sign of coronary arterial lesion.

An atrial flutter was detected on day 20 of the illness (Figure 1(c)). The atrial rate was 310 bpm, with a resultant 2 : 1 to 4 : 1 atrioventricular conduction block. Cardioversion was performed, as the atrial flutter continued for >18 hours. The patient was subsequently administered flecainide to prevent atrial flutter; however, this treatment was discontinued due to a significant sinus arrest of 5.7 seconds (Figure 1(d)). Sinus arrest gradually attenuated and was alleviated 3 days later.

**Figure 3:**
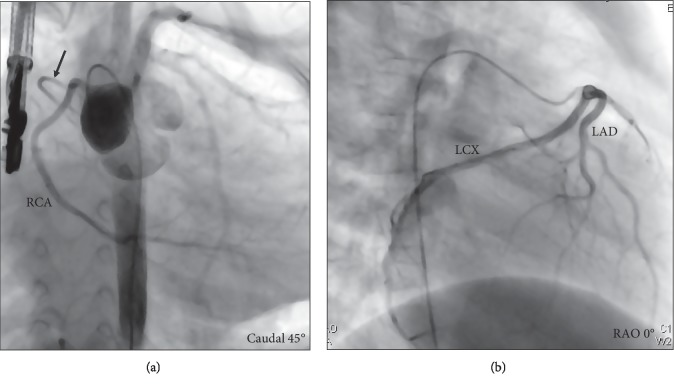
Coronary angiography (on day 25 of the illness). The arrow indicates sinus node branch from the right coronary artery. There was no dilatation or stenosis of the coronary arteries.

**Figure 4:**
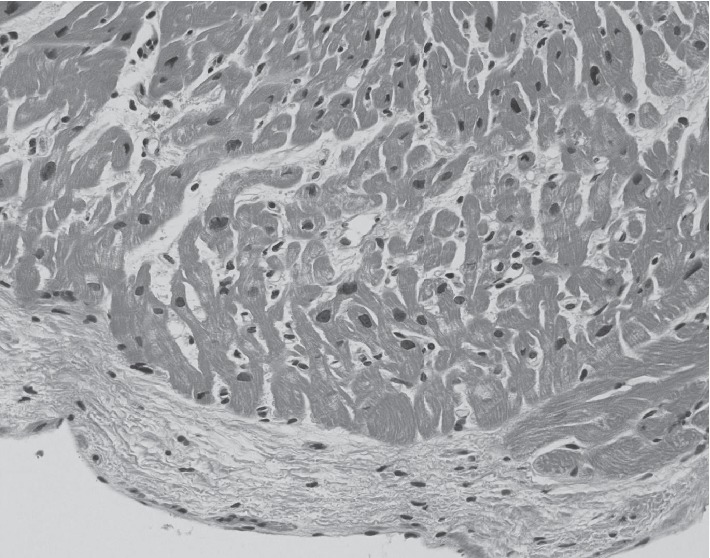
Histopathology of myocardium (on day 25 of the illness). There was no finding of the infiltration of the inflammatory cells. The myocardial cell had variation in the size and irregular adhesion, and fibrosis was seen between the muscular fiber. These were not findings of the acute phase but chronic stage, which were delivered by the pressure overload of the right ventricle due to the pulmonary arterial stenosis. Hematoxylin eosin, ×160.

Coronary angiography performed on day 25 of the illness demonstrated no dilatation or stenosis of the coronary arteries, and the sinus node artery was seen in normal origin from the right coronary artery (Figure 3). A myocardial biopsy was simultaneously performed. Myocardial cells exhibited variation in size and irregular adhesion, and fibrosis was detected between the muscular fibers (Figure 4). These findings may be attributed to the pressure overload on the right ventricle due to pulmonary arterial stenosis. Pathological findings of myocardium detected no evidence of myocarditis because the timing of biopsy was late to observe the findings of the acute phase of myocarditis.

Cerebrospinal fluid and serum cytokine data at admission are presented in Table 1. CSF and serum levels of interleukin (IL)-2 and IL-10 were elevated, while the CSF and serum levels of IL-6 and interferon-*γ* were significantly increased. The CSF level of IL-6 was two times higher than the serum level of IL-6. The patient was discharged without significant sequelae of cardiac and central nervous system complications.

## 3. Discussion

In the acute stage, about 10% of patients of KD show neurological manifestations such as irritability and lethargy which are caused by encephalitis [1]. Inflammation of the microvessels of the entire body can be observed by pathological inspection and by magnetic resonance imaging during this period, and this inflammation of the microvesseles causeencephalitis [7]. Histopathological findings of the central nervous system (CNS) of KD patients with encephalitis revealed aseptic chorio and/or leptomeningitis [8]. The inflammatory cells in the CNS were mainly lymphocytes and mononuclear cells, and such were corresponding to the findings in the CSFs [8] although our case did not have pleocytosis of the CSF. On the other hand, several reports revealed that IL-6 and tumor necrosis factor-*α* levels are extremely high in both the CSF and serum or in the CSF of KD [9, 10]. And, Aiba et al. reported that in addition to peripheral T cells, IL-6 is produced by oligodendrocytes, astrocytes, and other glial cells in the brain, and this IL-6 induces encephalitis [11]. We infer that IL-6 was associated with the encephalitis observed in the present patient, as the CSF and serum levels of IL-6 were extremely high.

It has previously been reported that although sinus node disturbances induced by KD are not easily identified by body surface electrocardiogram, they are prolonged to a high rate if they are assessed by an electrophysiological study [12]. Previous studies have suggested that vasculitis of the sinus node artery causes disturbance in blood perfusion to the sinus node, resulting in a disturbance in sinus node function [12]. Other studies have indicated that the inflammatory cells infiltrate the sinus node and neighboring ganglion; therefore, inflammation spreads to the sinus node [6].

Concerning the present patient, it is important to consider the influence of the previous operation for the congenital heart disease. Arrhythmias have been detected in 60/624 (9.6%) patients who underwent the arterial switch operation for transposition of the great arteries, and sick sinus syndrome (SSS) was detected in 6/624 (0.96%) patients. Furthermore, approximately half of all these cases developed SSS >5 years after the arterial switch procedure [13]. We hypothesize that subclinical sinus node dysfunction was induced by the arterial switch operation for the Taussig–Bing anomaly, and the disturbance of the blood perfusion of the sinus node and the inflammation of the sinus node occurred during the acute phase of KD, which in combination induced SSS.

Following transposition of the great arteries in patients who underwent arterial switch operation, all episodes of syncope were caused by complete atrioventricular block or SSS [13]. Pacemaker implantation (PMI) must be considered if SSS or complete atrioventricular block was not transient. Even though the present case met the criteria of PMI during the acute stage of KD, sinus node function returned to normal and special treatment was not required for arrhythmia after recovery from KD. However, the possibility of progression to SSS as the sequelae of inflammation and the influence of arterial switch operation in the future cannot be denied.

In conclusion, the present study described a case of KD accompanied by encephalitis and various types of arrhythmias during the acute phase. Both KD-induced vasculitis and arterial switch operation may have influenced the sinus node dysfunction. Although normal sinus node function was recovered, the possibility of progression to SSS should be considered. Therefore, it is essential the patient be continuously observed.

## Figures and Tables

**Table 1:** Cerebrospinal fluid and serum cytokine data.

	IL-2	IL-4	IL-6	IL-10	IFN-*γ*	TNF
CSF (normal range) (pg/ml)	5.81 (<2.6)	4.00 (<6.6)	234.27 (<6.2)	3.55 (<2.8)	156.88 (<7.1)	2.12 (<3.5)
Serum (normal range) (pg/ml)	3.78 (<3.9)	4.26 (<3.8)	126.63 (<9.5)	7.76 (<6.8)	658.25 (<21.1)	3.03 (<3.9)
